# Perceptual awareness and active inference

**DOI:** 10.1093/nc/niz012

**Published:** 2019-09-10

**Authors:** Thomas Parr, Andrew W Corcoran, Karl J Friston, Jakob Hohwy

**Affiliations:** 1 Wellcome Centre for Human Neuroimaging, Institute of Neurology, University College London, Institute of Neurology, 12 Queen Square, London, UK; 2 Cognition & Philosophy Laboratory, Department of Philosophy, Monash University, Melbourne, Australia

**Keywords:** active inference, awareness, Troxler fading, binocular rivalry, Bayesian

## Abstract

Perceptual awareness depends upon the way in which we engage with our sensorium. This notion is central to active inference, a theoretical framework that treats perception and action as inferential processes. This variational perspective on cognition formalizes the notion of perception as hypothesis testing and treats actions as experiments that are designed (in part) to gather evidence for or against alternative hypotheses. The common treatment of perception and action affords a useful interpretation of certain perceptual phenomena whose active component is often not acknowledged. In this article, we start by considering Troxler fading – the dissipation of a peripheral percept during maintenance of fixation, and its recovery during free (saccadic) exploration. This offers an important example of the failure to maintain a percept without actively interrogating a visual scene. We argue that this may be understood in terms of the accumulation of uncertainty about a hypothesized stimulus when free exploration is disrupted by experimental instructions or pathology. Once we take this view, we can generalize the idea of using bodily (oculomotor) action to resolve uncertainty to include the use of mental (attentional) actions for the same purpose. This affords a useful way to think about binocular rivalry paradigms, in which perceptual changes need not be associated with an overt movement.

## Introduction

Bayesian accounts of brain function (Knill and Pouget 2004; Doya 2007; De Ridder *et al.* 2014) rely upon the idea that the brain utilizes an internal generative model (Von Helmholtz 1867) of its environment to make predictions about incoming sensory data, and that it adjusts this model when these predictions are not fulfilled (Rao and Ballard 1999; Friston and Kiebel 2009). This view formalizes the notion of perception as hypothesis testing (Gregory 1980) and facilitates the extension of this to the metaphor of the brain as a scientist who actively performs experiments (actions) to disambiguate between competing models of the world (Friston *et al.* 2015). Although this perspective has been successful in understanding a range of behavioural, anatomical and neurophysiological findings (Friston *et al.* 2017a; Parr and Friston 2018), some phenomena appear at odds with the idea that perception is inference. Here, we focus upon two such phenomena in which percepts appear to deviate from what we might intuitively expect from optimal inference. Specifically, both involve the perception of change over time, despite static sensory input. These include Troxler fading, in which fixation in the centre of an unchanging stimulus is associated with the percept of fading of peripheral stimuli (Troxler 1804); and perceptual (e.g. binocular) rivalry, in which awareness alternates between two distinct percepts. We argue that these percepts can be framed as optimal inferences [in line with the complete class theorems (Wald 1947)] and that their apparent sub-optimality affords an opportunity to explore the sorts of prior beliefs that are necessary to make these Bayes optimal [Bayes optimality is quantified in terms of Bayesian model evidence (i.e. how probable data are under a given model). This is the quantity approximated by negative free energy. Note that, because free energy is a functional of prior beliefs, the behaviour that maximizes model evidence will differ depending upon those beliefs].

**Figure 1.**
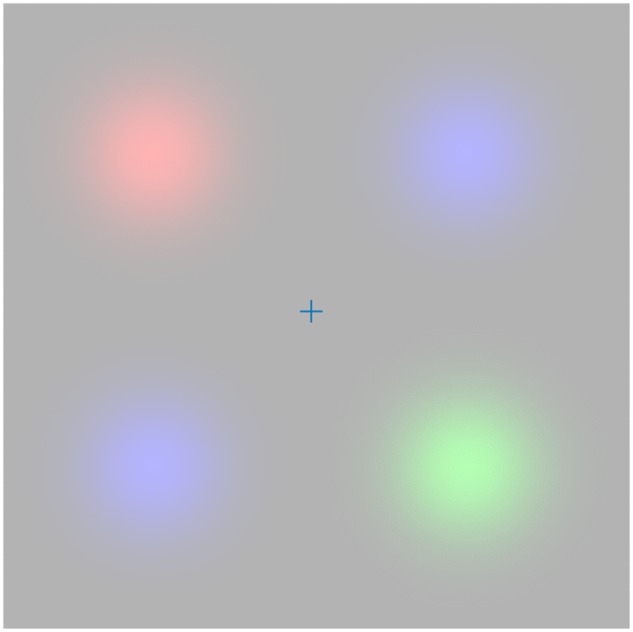
Troxler fading. The image shown here is a simple example of the sorts of stimuli used to induce Troxler fading. During free (saccadic) exploration of this image, four blurred circles are visible, in various colours. On fixating on the cross in the centre of this image, the coloured stimuli gradually fade until they match the grey of the background. Once free exploration is resumed, the percepts of the colours are reinstated. This provides an interesting example of the role of action in perception, as the presence or absence of action leads to dramatically different percepts.

This sort of approach has been employed with great success to aid understanding our susceptibility to a range of visual illusions (Geisler and Kersten 2002; Weiss *et al.* 2002; Brown and Friston 2012; Brown *et al.* 2013). In what follows, we make use of similar ideas but place a special emphasis upon active engagement with the sensorium. The importance of action in perception is simply demonstrated in Fig. 1, which shows a stimulus of the sort that can induce Troxler fading. On extended fixation of the central cross, the colours in the periphery appear to fade away. The colours immediately re-appear upon resumption of normal exploratory eye movements. This simple example illustrates the need to consider the *active* inferential processes that underwrite perceptual awareness. In the following sections, we first provide a brief overview of the generic aspects of active inference and then turn to the specific generative model used to illustrate the importance of action in perception; under beliefs that the world may change in an unpredictable way. We then apply the conclusions drawn from these simulations to try to explain perceptual (binocular) rivalry. A comprehensive evaluation of all previous empirical and experimental data is beyond the scope of the present paper, which presents a starting point for further research. Ultimately, the theoretical accounts proposed here represent hypotheses as to the generative models and, implicitly, computational mechanisms that underwrite these perceptual phenomena. As such, they must be answerable to empirical data. To facilitate this, we highlight some of the predictions that arise from this approach that we hope to test through psychophysical and neuroimaging experiments.

## Active Inference

The ideas outlined in the introduction may be formalized by appealing to active inference. This is a theoretical perspective on brain function that frames action and perception as ‘self-evidencing’ (Hohwy 2016) or free energy minimizing processes. Another way of putting this is that these are inferential processes, with perception representing inferences about the causes of sensory data, and action planning as a process of inference about ‘what I am going to do next’. A simple way to motivate this perspective is to start from the idea that actions (*u*) change the external world such that some function of sensory observations (*o*) is optimized (Friston 2013):

(1) $$u=\underset{u}{\arg\;\max}L(o(u))$$

This premise is essentially a restatement of the principle of homeostasis, as it says that actions correct deviations in observed values (where observations encompass both external and internal environments), returning them to their optimum values. It also underwrites a range of theoretical accounts of behaviour, where creatures are assumed to maximize the ‘value’ or ‘utility’ of the data they observe. Given that this function of observations may be maximized, we can treat this as an un-normalized log probability (often referred to as ‘model evidence’):

(2) $$\begin{array}{l} u=\underset{u}{\arg\;\max}\;\ln\;P(o(u))\\ P(o(u))=\sum_{\pi ,s}P(o(u),s,\pi ) \end{array}$$

It is this expression that justifies the self-evidencing perspective, as the drive to optimize sensations has been re-expressed as a drive to seek those sensory data that afford greater evidence to one’s model of the world. Evidence is used here in the technical sense, as a marginal likelihood (probability of data under a model). This is made explicit in the second line of Equation (2), in which the evidence is given as the marginal of a distribution that accounts for the generation of observations by hidden states (*s*) of the world, and the policy (course of action) pursued (*π*) by the creature in question. From a variational perspective [variational methods originated in physics as a way of turning a difficult integration problem into an optimization problem Feynman, R. P. (1998). In the context of variational inference, this is used to convert the problem of summing over all unknown variables into an optimization of a quantity called free energy (in statistics and machine learning, negative free energy is sometimes referred to as the evidence lower bound or ‘ELBO’)] we can re-write Equation (2) in terms of a free energy functional (*F*) (Dayan *et al.* 1995; Beal 2003; Friston *et al.* 2010):

(3) $$\begin{array}{c} u=\underset{u}{\arg\min}F\\ F=E_{Q}[\ln Q(s,\pi )-\ln P(o,s,\pi )]\\ =-E_{Q}[\ln P(o,s,\pi )]-H[Q(s,\pi )]\\ =-\ln P(o)+D_{\mathrm{KL}}[Q(s,\pi )||P(s,\pi |o)]\\ \approx -\ln P(o)\Leftrightarrow Q(s,\pi )=\underset{Q}{\arg\min}D_{\mathrm{KL}}[Q(s,\pi )||P(s,\pi |o)] \end{array}$$

**Figure 2.**
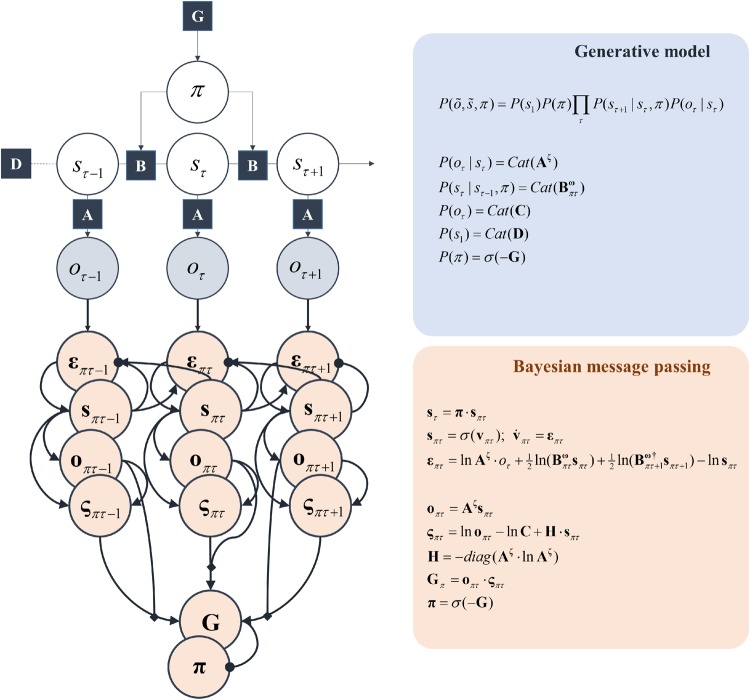
Markov decision process. The schematic above shows a factor graph representation (blue) of a (partially observed) Markov decision process. The circles represent variables, whereas the squares are the factors of the generative model that describe the probabilistic relationship between these variables. The panel on the upper right specifies the form of these relationships. Specifically, **A** is a probability (likelihood) distribution that specifies the probability of an observation (*o*) given a hidden state (*s*). The initial state is given by a probability vector (**D**), and the subsequent states in the sequence depend upon the probability transition matrix (**B**). This expresses the dependence of each state on the previous state in the sequence, and upon the policy (course of action) pursued (*π*). The policy itself is determined by a prior belief that the most probable policies are those that are associated with the lowest expected free energy (**G**), that itself depends upon prior preference (**C**). The lower (pink) panels show the Bayesian inversion of the model above, taking the outcomes it generates, and computing posterior beliefs about the states (**s**), predictive beliefs about future outcomes (**o**) and beliefs about the policy being pursued (**π**). These computations are expressed in terms of auxiliary variables playing the role of prediction errors. These are the free energy gradients (**ε**), and the expected free energy gradients (**ς**). The transition and likelihood probabilities are each equipped with a superscripted term (**ω** and **ζ**, respectively). These represent inverse temperature (or precision) terms that we will use to quantify the (inverse) uncertainty inherent in these probabilities. Note that this Bayesian message passing relies upon local interactions [specifically, marginal message passing (Friston *et al.* 2017a; Parr et al. 2019)], very much like those between neurons in a network. The notation *Cat* means a categorical distribution, while *σ* is a softmax (normalized exponential) function. Note that **ω**, **ζ** and **C** appear in the panels on the right, but do not appear in the factor graph. The reason for this is that the factor graph formalism we have used specifically describes the relationship between random variables (i.e. those things about which we have probabilistic beliefs). Both **ω** and **ζ** are parameters of distributions, but we do not model explicit beliefs about them. The influence of **C** is more subtle. This only has an effect via the expected free energy, so may be thought of as a constituent of the **G** factor-node.

This equation says that when our beliefs (*Q*) approximate the (posterior) probability of states and policies (i.e. sequences of actions) given sensory data (observations), minimizing free energy approximates maximization of model evidence. This emphasizes the (approximately) Bayesian inferential processes implicated in self-evidencing. In the following, we equate perception with optimization of beliefs about states and planning with optimization of beliefs about policies. These rest upon a generative model of the form shown in Fig. 2, in which states cause observations, and themselves are caused by states at previous times, depending upon the policy pursued. Appealing to the same ideas outlined in this section, we set the prior belief about policies such that those policies with the smallest expected free energy (*G*) are considered more probable (Friston *et al.* 2017b,c).

(4) $$\begin{array}{c} \ln P(\pi )=-G(\pi )\\ G(\pi )=E_{\tilde{Q}}[\ln Q(s|\pi )-\ln P(o,s)]\\ =E_{Q}[H\underset{\left(1\right)}{\left[P(o|s)\right]}]-\underset{\left(2\right)}{H[Q(o|\pi )]}-E_{\tilde{Q}}\underset{\left(3\right)}{\left[\ln P(o)\right]}\\ \tilde{Q}(o,s|\pi )=Q(s|\pi )P(o|s) \end{array}$$

In Equation (4), the expected free energy is expressed in two ways. The first emphasizes the similarity between it and the free energy of Equation (3). The second splits this into three terms with distinct interpretations. Term 1 expresses the ambiguity of a state-outcome mapping. The greater this term, the lower the fidelity of this mapping. Term 2 expresses the uncertainty in predicting the outcome that would be observed conditioned upon the policy (i.e. the entropy of posterior predictive beliefs). Together, terms 1 and 2 express the amount of resolvable uncertainty associated with a policy (Lindley 1956). Term 3 may be thought of as encoding preferences about the sorts of outcomes that will be sought (Friston and Ao 2012). In summary, the first two terms of the expected free energy favour the performance of experiments to disambiguate between alternative hypotheses about the world, whereas the final term biases this towards seeking out those data consistent with one’s favoured hypothesis. The novel aspects of the treatment in this article rest upon this (expected free energy) functional. As we will see later, it is this that drives the (mental or motoric) active selection of data to resolve the uncertainty that underwrites visual sampling (and precludes stimulus fading) and that gives rise to the perceptual transitions in binocular rivalry. This builds upon previous accounts of these phenomena that focus on passive perceptual processing in the presence of ambiguous data.

In addition to showing the form of *P*, the generative model, Fig. 2 illustrates how a neuronal network could make inferences about this generative model through gradient descents on the variational free energy, while selecting policies that minimize the expected free energy (Friston *et al.* 2017a, d).

## Action in Perception

**Figure 3.**
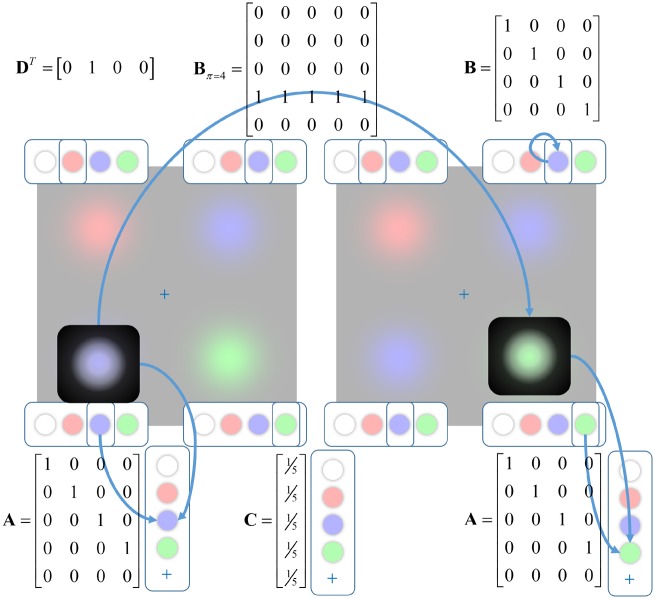
Troxler generative model. This schematic illustrates the form of the generative model used to simulate Troxler fading. It includes five hidden state factors representing the colour of the stimulus (shown here in the rows at each corner of the image) at each location and the current fixation location (highlighted in black). The prior probabilities (**D**) for each hidden state are deterministic, with fixation location starting in the centre, and the colours as indicated above. The only outcome modality is visual (illustrated in the columns below each image), and the mapping from the hidden state associated with a fixated location is shown in the **A**-matrices here. This is shown for fixation on the lower left (left image) and the lower right (right image). Note that there is only one visual input at any one time, so vision depends upon eye position. The **B**-matrices specify transitions between hidden states over time. For the stimulus hidden states, these are identity matrices (see upper right). For the fixation location, the transitions depend upon the selected action. Here, we show an example for action four, for which all states transition to state four. This is illustrated explicitly in the transition from the left to the right image. Preferences (**C**) for each outcome are set to be uniform. Note that this model is formally identical to that pursued in Parr and Friston (2017c) to investigate the salience of different locations under different beliefs about their precisions, but with a different semantic interpretation. The likelihood and transition matrices here are equipped with precision terms to convert them to the probabilities of Figure 2 as follows: $A^{\zeta }=\sigma (\zeta \ln(A+e^{-4}))$, $B^{\omega }=\sigma (\omega \ln(B+e^{-4}))$. The precision associated with the transition matrix for the control (fixation) state factor is always treated as infinite.

To illustrate the importance of action in perception (Martinez-Conde *et al.* 2004; Zimmermann and Lappe 2016; Parr and Friston 2017a; Bruineberg *et al.* 2018), we constructed a generative model of the sort that might be used by the brain in visual scene construction (Mirza *et al.* 2016). Similar models have been validated empirically in human subjects (Mirza *et al.* 2018). The form of the model is shown in Fig. 3. This comprises five types of hidden state: four that indicate the colour shown in each quadrant, and one that represents fixation location. As shown in the lower part of the figure, when fixating on the lower left quadrant (left image), the visual outcome is determined by an identity (i.e. deterministic one-to-one) mapping from the hidden state indicating the colour in this location (i.e. the hidden states map directly to their associated outcomes). On performing a saccade to the lower right quadrant (right image), the visual outcome is determined by an identity mapping from the hidden state representing the lower right quadrant (**A**). This structure means that the subject may choose which of the hidden states to interrogate by selecting an eye movement. The eye movements depend upon which transition matrix (**B**) is selected. The matrix shown in the centre at the upper part of the image illustrates the form that this takes when policy 4 is selected. Whichever position is initially fixated (columns), there is a probability of 1 that location 4 is fixated at the next time-step (rows). This is illustrated in the figure by the move from the lower left to the lower right quadrant.

The transitions associated with the stimuli at each location are set to be identity matrices. This ensures that the stimulus is static throughout time. In the generative model (the beliefs the brain has about how the data were generated), we equip these matrices with a precision (inverse temperature) (**ω**) that permits the possibility that the stimuli may change from moment to moment. A similar parameterization is used for the likelihoods (with precision **ζ**). The smaller these precisions are, the more uncertain the associated probabilities. The **C**-matrix expresses preferences over outcomes, here set to be uniform (i.e. no outcome is preferable relative to any other). Beliefs about initial states (**D**) are the same for the generative model, and the process generating the simulated data. These ensure the initial states of each stimulus are known, and that the initial fixation location is at the fixation cross. The idea was to initialize with the beliefs we might have following free exploration, so that the start of the simulation corresponds to the point at which we have been asked to fixate or to continue exploring. This makes very little difference for the latter, where similarly confident beliefs are maintained throughout. However, had we started with completely uncertain beliefs at the point of the instruction to maintain fixation, we would have had no precise percept to fade away. Although our synthetic subjects start with veridical prior beliefs, the uncertainty in the transitions (1/**ω**) ensures that, in the absence of new data, the uncertainty associated with these beliefs will increase with time since the start of the trial [c.f. ‘distrusting the present’ (Hohwy *et al.* 2016)].

**Figure 4.**
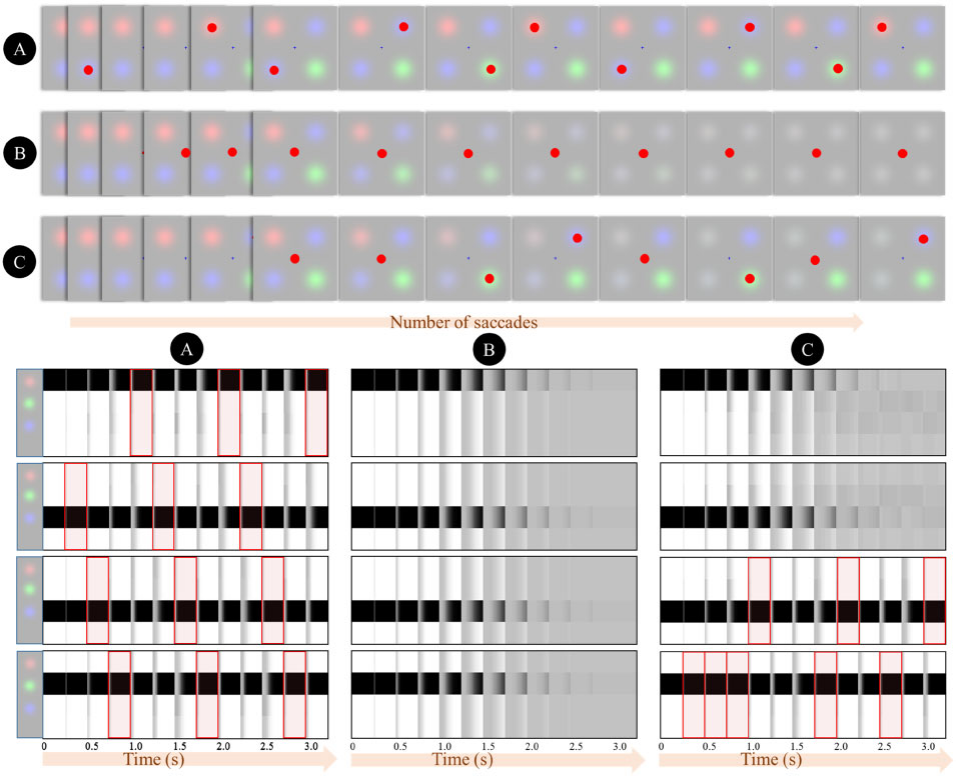
Simulated Troxler fading. The upper plots illustrate the percepts and saccadic patterns obtained by solving the equations of Figure 2 for the model of Figure 3. The percepts are obtained by weighting the images by their posterior probabilities (which are plotted in the lower plots). (A) It shows the case in which saccadic exploration ensures the maintenance of the image over time. (B) It shows the result of introducing a preference (**C-**vector Figure 3) for the fixation cross. This results in a failure to explore, and the gradual fading of the percept. (C) It depicts a neglect-syndrome, in which a prior belief that rightward saccades are more probable than leftward saccades gives rise to an asymmetrical exploration, leading to an accumulation of uncertainty about the left side of space. The lower parts of this Figure show the beliefs at each time-point about each of the four stimulus locations. Each plot illustrates beliefs about which stimulus (if any) is present at a given location. From top to bottom, these are upper left, lower left, upper right, lower right. For each of these plots, there are four rows, corresponding to the different hidden states that might be present at those locations. Within these rows, black represents a probability of one that this is the stimulus (hidden state) at this location, whereas white represents a probability of zero. Intermediate shades indicate uncertainty about the hidden state. The red highlights indicate which location is fixated at any given time. For time-steps with no highlight, the fixation cross was fixated.

Crucially, because the likelihood is equivalently precise for each quadrant, and because the preferences are uniform, the only component of the expected free energy that varies between saccades to each location is the entropy of posterior predictive beliefs [term 2 in Equation (4)]. This drives the behaviour illustrated in Fig. 4A. Inspection of the lower plots that illustrate beliefs throughout the trial reveals that on saccadic fixation (highlighted in red), there is minimal uncertainty. Over successive time-steps, the uncertainty accumulates, leading to more grey (probabilities between 0 and 1) in the belief plots. At each time, the maximally uncertain location is selected for the next saccade in accordance with term 2 in Equation (4). Another way of looking at this is that selection of policies to minimize expected free energy naturally gives rise to an ‘inhibition of return’ phenomenon (Posner *et al.* 1985; Klein 2000), whereby recently fixated locations lose their salience (Parr and Friston 2019) relative to those locations in which something might have changed (Von Helmholtz 1867; Hohwy 2012).

Figure 4B illustrates what happens when we instruct our simulated participant to maintain fixation on the central cross. This instruction is given by increasing the preference for seeing the cross over all other possible outcomes (encoded in **C**). The lack of exploratory eye movements here means that the accumulating uncertainty cannot be counteracted, and beliefs about the stimuli rapidly become uniform. This is illustrated in the upper plot as a fading of the peripheral stimuli. Under this model, the dissipation of the percept can be seen as compliance with Jaynes’ maximum entropy principle (Jaynes 1957), which says that the best inference is that which has the maximum Shannon entropy (uncertainty) under the constraints imposed by observations. This imperative is formalized in free energy minimization, as the free energy may be expressed in terms of an ‘energy’ minus an ‘entropy’ [third line of Equation (3)]. As data contributes only to the ‘energy’ term, an inability to seek out new data favours the ‘entropy’ term.

Figure 4C shows a more selective deficit, in which we have introduced a bias towards rightward saccades. This device has previously been used to replicate the deficits of exploratory eye movements associated with visual neglect (Parr and Friston 2017b). Neglect is a neuropsychological syndrome, normally following pathological insult to the right cerebral hemisphere, characterized by an impaired awareness of stimuli on the left side of space (Halligan and Marshall 1998). This condition is of great importance to the issue of action in perception, as a behavioural characteristic of neglect is a diminished frequency of saccades to the left (Husain *et al.* 2001; Fruhmann Berger *et al.* 2008; Karnath and Rorden 2012). The simulations here illustrate a plausible interaction between these behavioural and perceptual aspects of neglect, as uncertainty accumulates in those regions that are not fixated.

**Figure 5.**
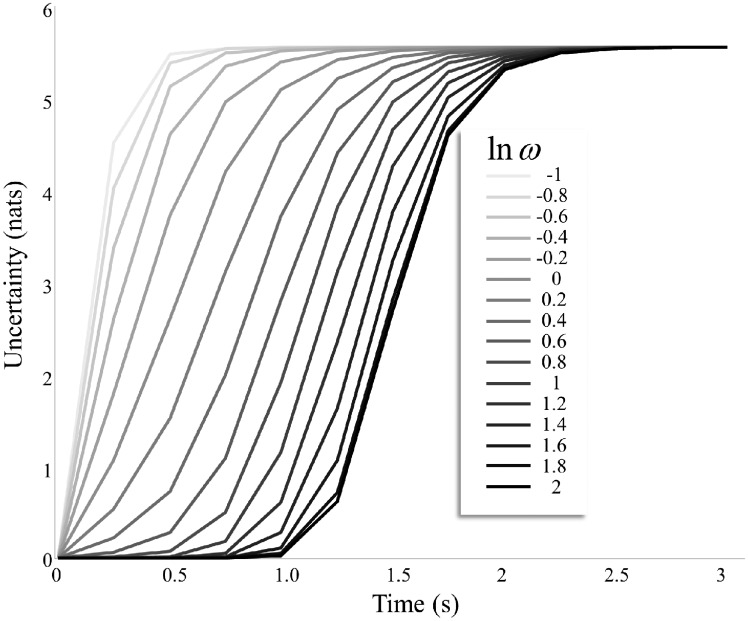
Transition precision. This plot illustrates how the time until the images fade depends upon prior beliefs about the precision of transitions. This is expressed as the accumulation of uncertainty over time, where uncertainty is the Shannon entropy summed over posterior beliefs about each stimulus. All lines converge upon maximum uncertainty, corresponding to the completely faded stimuli at the end of row B in Figure 4. As the precision of transitions (**ω**) is increased, the time it takes for the stimulus to fade increases. This affords an opportunity for empirical investigation, as it suggests the precision of beliefs about transitions (relative to some reference) should be estimable from the time it takes for a percept to fade during fixation of a Troxler stimulus.

Figure 5 illustrates an important feature of the model we have described. This shows the accumulation of uncertainty for the simulation of Fig. 4B in terms of the Shannon entropy of these beliefs over time. As beliefs about the precision of transitions increases, the time for the entropy to reach its maximum is increased. If we equate the maximum entropy with the point at which the image fades, it should be possible to estimate, from experimental data, the precision a given individual ascribes to transitions. This makes a strong empirical prediction, in that the time taken for the image to fade should be inversely related to measures of volatility (the imprecision of dynamics) that can be estimated through other experimental paradigms (Mathys *et al.* 2014; Marshall *et al.* 2016; Lawson *et al.* 2017). Given the association between noradrenergic signalling and precision, it may also be possible to estimate these parameters from pupillary data (Koss 1986) on an individual basis (Vincent *et al.* 2019). This additionally predicts that pharmacological manipulation of catecholamine signalling would systematically change the time until an image fades. The decay of precise posterior beliefs over time rests upon (beliefs about) the changeability of the environment (Hohwy *et al.* 2016) or, more exactly, the precision of the transition probabilities that lead from one state to the next. This resonates with the use of ‘fatigue’ terms in previous models of binocular rivalry (Dayan 1998). However, while this was previously incorporated into the inference dynamics explicitly, we find that the same sort of decay emerges naturally from a generative model that entails stochastic transitions. In the next section, we translate the key features of the model used above to the setting of binocular rivalry.

A common explanation for Troxler fading is that cells in the peripheral retina fatigue (or ‘adapt’) on sustained activation (Martinez-Conde *et al.* 2004). Unless we assume perceptual awareness arises directly from the retinal activity, this explanation is highly consistent with the explanation on offer here. It implies that, during sustained fixation, little precise information from outside the fovea is propagated to the brain. However, it is well known that the precision of data from the peripheral retina is limited even in free visual foraging. Our experience of a visual scene is typically thought of as an internally generated percept [or inference (Von Helmholtz 1867)], informed by the spatially limited data obtained from sequences of fixations. The implication here is that the absence of precise data should not lead to an imprecise inference about a previously observed stimulus, unless the generative model used to make these inferences allows for stochastic dynamics that ensure uncertainty accumulates over time. Following this line of reasoning, evidence in favour of cortical involvement in these processes is unsurprizing (Lleras and Moore 2001; Hsieh and Tse 2006; Kanai *et al.* 2008).

## Attention as Action

In this section, we move from Troxler fading, in which overt actions are crucial in maintaining a percept, to binocular rivalry, in which no overt action takes place. This is the same move that motivates the premotor theory of attention (Rizzolatti *et al.* 1987). Our aim is to show that exactly the same principles that give rise to Troxler fading also offer a plausible account of rivalry.

Binocular rivalry occurs in the presence of data that could be explained by one of two (or more) hypotheses (Brascamp *et al.* 2018). A common way to induce this is to present different images to each eye. If we take the view that the brain is a passive recipient of sensory data, we might expect the resulting percept to be some blend of these explanations (Clark 2018), as it is impossible to disambiguate between these with any degree of certainty. However, the resulting percept actually alternates between the two hypotheses. Although this phenomenon is difficult to account for from a ‘Bayesian brain’ perspective, it becomes much simpler once we incorporate action. This is because pursuing an action forces us to commit to interrogating one dimension (factor) of a hypothesis at a time (Hohwy 2013), just as in the Troxler fading example above. In that case, the spatial configuration of stimuli enforced the constraint that only one stimulus could be fixated at any one time. However, beliefs about the changeability of the world drove alternations in which location was fixated at any one time. Here, we generalize this to the notion of an attentional (mental) action (Limanowski and Friston 2018) directed to one set of features or another. In other words, attention may be employed to resolve uncertainty about the presence or absence of one sort of stimulus or the presence or absence of another. These can be seen as two alternative dimensions in a hypothesis space, as both could be present, both absent, or one present and the other absent. We appeal to the same idea that the world is changeable, such that those states informed by unattended features rapidly accumulate uncertainty (and consequently epistemic value), ensuring an alternation of attention. This is highly consistent with accounts of rivalry as an attentional phenomenon (Leopold and Logothetis 1999; Zhang *et al.* 2011). It also echoes previous ideas concerning the accumulation of uncertainty for the suppressed stimulus (Hohwy *et al.* 2008), but supplements this idea with an appeal to the accumulation of epistemic value this entails.

The generative model used for this section has the same Markov Decision Process form as that of the previous section. There are two outcome modalities: the presence or absence of visual data consistent with the letter ‘L’, and the presence or absence of visual data consistent with an ‘R’. In the simulated environment (generative process), these are generated by two hidden state factors through identity mappings. The generative model (our synthetic subject’s beliefs about the environment) is additionally equipped with an attentional state, which plays a similar role to that of the fixation location in the previous section. Unlike the fixation location, this has no influence over the data that are actually available to the subject. Instead, it modulates their *beliefs* about the fidelity (precision) of the mapping from the ‘R’ state to the ‘R’ outcome, and from the ‘L’ state to the ‘L’ outcome. When attending to ‘R’, the ‘L’ outcome is assumed to be randomly (imprecisely) generated and is uninformative about the ‘L’ state, and vice versa when attending to ‘L’. As before, the ambiguity associated with each attentional policy is equivalent when summed overall outcomes. This means that the drive to select a given policy is determined by the predictive entropy, or ‘how uncertain am I about what I would see if I attended to that?’ Note that this generative model does not preclude an inference that both stimuli are present simultaneously. However, it would be relatively simple to incorporate inferences about the location of each stimulus in space, where the prior probability that they occupy the same location is zero.

**Figure 6.**
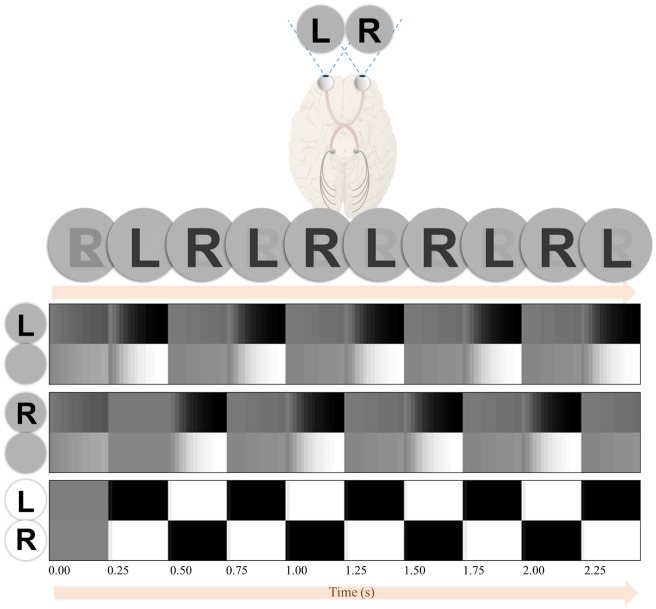
Simulated binocular rivalry. The upper part of this figure shows a simple schematic of a binocular rivalry paradigm. The image presented to each eye is different (here shown simply as an ‘L’ and an ‘R’). This sets up two competing percepts. The row of circles below this shows the posterior beliefs about the stimuli, with the stimulus intensity represented as a monotonic function of its posterior probability. The plots below show beliefs about the presence or absence of the ‘L’, the presence or absence of the ‘R’, and whether attention is directed towards the ‘L’ or ‘R’ features. Note that, when attention is directed towards ‘L’, this induces a belief that the ‘L’ is present, but increases the uncertainty about whether the ‘R’ is present or absent and vice versa. The attended features are consistently those for which the uncertainty was greatest at the previous time-step, just as with the choices of fixation location in the Troxler fading simulation. Although changes in percept coincide with the changes in attentional focus, the two are not equivalent. The former are changes in posterior beliefs, and are consequent upon the changes in precision assumed under alternative attentional choices. The subtlety of this distinction, and the reciprocal causation (attentional choices depend upon posterior beliefs) between the two may underwrite debates about the relationship between attention and awareness, e.g. (Lamme 2003).

**Figure 7.**
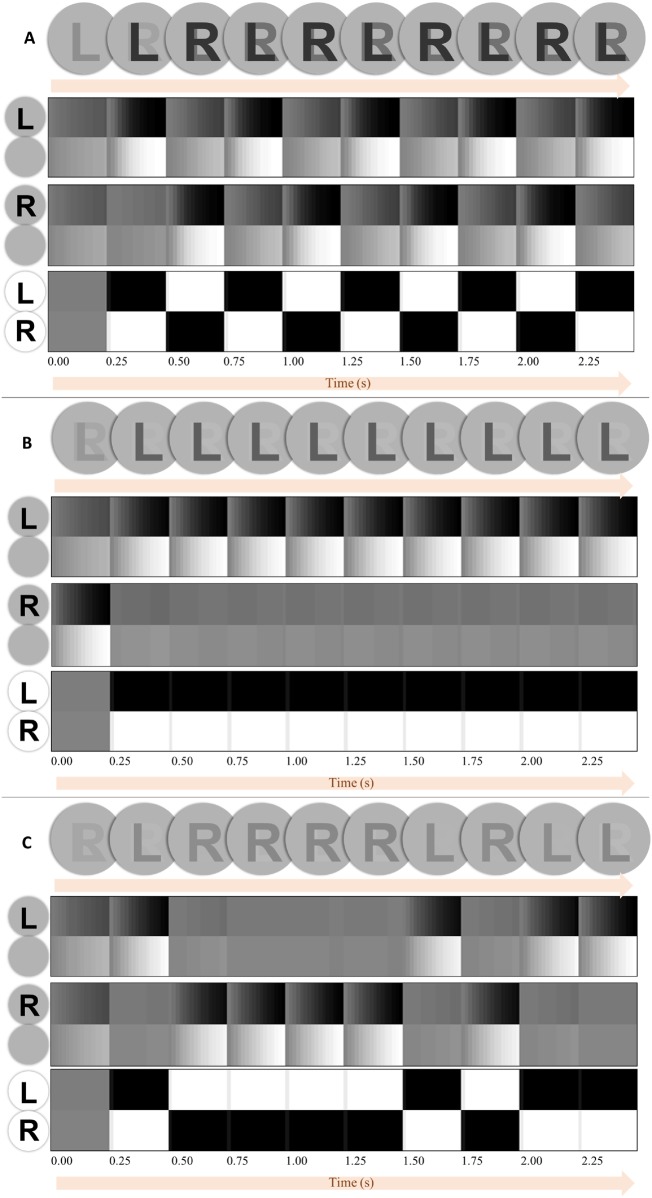
Precisions. This figure shows three special cases of the generative model used above, to provide some intuition as to the behaviour of the simulations. The upper plots (A) show the same set-up as in Fig. 6, with the same generative process giving rise to the data. However, we have adjusted the beliefs of our synthetic subject such that they estimate the precision of transitions to be higher (i.e. a less changeable environment). Although the perceptual switches still continue, the percept does not change as dramatically, as beliefs about the previously attended stimuli persist for a greater time. In the limiting case in which a subject believes the world does not change at all the percept would appear as a mixture of the two stimuli that does not change over time (note that a minority of people do indeed report such fused percepts, but that this leads to their exclusion from standard rivalry studies). In other words, beliefs about the precision of transitions in a person’s generative model may underwrite their susceptibility to rivalry. The middle plots (B) in this figure illustrate the influence of the likelihood precision associated with each stimulus. Here, we have decreased the precision for both stimuli, the ‘R’ stimulus more than the ‘L’ stimulus. This alters the subject’s beliefs about the ‘noisiness’ of the two observations she could make, such that both are noisy, but the ‘L’ stimulus is more reliable. Experimentally, this sort of belief can be induced by changing the contrast of the image presented to each eye. Notably, our subject infers that the best policy is to attend only to the relatively unambiguous ‘L’ stimulus and to consistently ignore the ambiguous ‘R’. The lowest plot (C) shows the same manipulation as (B), but with a much subtler difference between the two precisions. This is just enough to break the symmetry between the two stimuli, but not enough to eliminate perceptual switching. The three examples in this figure illustrate that, even with exactly the same sensory data, different prior beliefs about the generation of these data can lead to dramatically different perceptual inferences. These differences offer the opportunity to investigate the distinct computational phenotypes that underwrite individual differences in perceptual experience.

Figure 6 shows the result of simulating this paradigm, using the same format to that of Fig. 4. Here, we present both an ‘R’ and an ‘L’ outcome. These could be presented to each eye independently, as in binocular rivalry paradigms. The alternation of the percept in the presence of static data illustrates the importance of internally generated dynamics in perception. Here, these dynamics emerge from the imperative to minimize expected free energy, under the prior belief that the world is volatile. Figure 7 shows the influence of various manipulations to the generative model on belief updating to provide intuition for its performance. The first is to change beliefs about the precision of transitions (Fig. 7A). This illustrates the importance of beliefs about the volatility of the world, as the slower rate of uncertainty accumulation under beliefs that transitions are static diminishes susceptibility to perceptual alternation. This additionally reinforces existing theories that argue for the importance of generative models about changing environments for binocular rivalry (Hohwy *et al.* 2008), and with those generative models that incorporate explicit decay terms (Dayan 1998) that implicitly model this change.

An increasing susceptibility to rivalry as expected environmental dynamics become less precise predicts that those psychopathologies that are associated with an impaired capacity to estimate volatility, such as schizophrenia and autism (Lawson *et al.* 2017; Palmer *et al.* 2017), should lead to more dramatic rivalrous percepts. These changes would not be seen in the frequency of perceptual alternation [although such changes have been reported (Robertson *et al.* 2013; Xiao *et al.* 2018)], but in the difference between the percept before and after a perceptual switch. The differences in the amount of belief updating at the time of a perceptual switch might manifest, and be measurable, using electroencephalography. For detailed accounts of the hypothesized relationship between electrophysiological measures and the belief updating described by active inference, please see Friston *et al.* (2017a, d). An important caveat is that there may be additional attentional deficits in conditions such as autism (Lawson *et al.* 2014; Palmer *et al.* 2015), which could confound this line of investigation and explain the mixed results in this field (Wykes *et al.* 2018). For example, autism has been associated with a failure of selective attention, or the capacity to attend away from irrelevant information. A failure to down-weight the gain of the non-attended stimulus in our simulated paradigm would eliminate any rivalrous phenomena, as the ability to selectively engage attention in an adaptive way underwrites the effects we have demonstrated. This failure of selective attention might counteract any increased susceptibility due to over-estimated volatility in the context of autism. An alternative to relying upon psychopathology would be to appeal to individual differences. The prediction here is that the amount of belief updating required following an attentional switch should be inversely related to the time taken for stimuli to fade in a Troxler paradigm (Fig. 5).

The second manipulation, shown in Fig. 7B, is an increase in the likelihood precision ascribed to one outcome modality compared with the other. In this example, we set the ‘L’ outcome to be less ambiguous than the ‘R’ outcome. This replicates a covert attentional (Rizzolatti *et al.* 1987; Sheliga *et al.* 1994) manifestation of the ‘Streetlight’ effect (Demirdjian *et al.* 2005), in which the superior quality of information afforded by attending to ‘L’ (or searching for one’s keys underneath a streetlight on an otherwise dark street) renders this an epistemically valuable policy to pursue. As such, perceptual switching is eliminated in favour of attention to the less ambiguous modality. This may be seen as analogous to the effect of altering the contrast of the image presented to one eye, which increases the perceptual dominance of the less ambiguous stimulus (c.f. Levelt 1965). A more extreme (but more common) example would be that of presenting the same image to both eyes such that there is no precise information about one hypothesis (that associated with the stimulus not presented), and nothing to be gained by attending to its associated features.

An interesting phenomenon that speaks to the attentional aspect of binocular rivalry is the slowing of transitions when attention is drawn away from the stimuli (Paffen *et al.* 2006; Alais *et al.* 2010). Under the framework employed here, this implies the addition of a third attentional policy. This would lead to a distribution of the time spent in each attentional focus among three (as opposed to two) alternatives, such that the frequency of selecting attention to ‘R’ or ‘L’ decreases. This implies a decrease in the frequency at which a percept transitions *to* ‘R’ or ‘L’.

Figure 7C illustrates an important point concerning the timing of perceptual switches. Under the idealized simulations in Fig. 6, these switches happen four times per second (i.e. on a theta cycle). This is consistent with evidence that such transitions are locked to this cycle (Doesburg *et al.* 2009). However, it is important to note that perceptual switches do not necessarily occur every 250 ms. By decreasing both likelihood precisions (e.g. by changing the stimulus contrast), but by very subtly different amounts, we can break the symmetry between the two stimuli that leads to the consistent and regular alternation of Fig. 6, to get the more irregular pattern of Fig. 7C. Note that this still involves switches that are locked to the theta cycle, but that a switch does not necessarily occur at every cycle.

The frequency of perceptual transitions has been studied extensively in relation to different experimental factors and clinical conditions (Robertson *et al.* 2013; Wykes *et al.* 2018; Xiao *et al.* 2018). However, the model presented here does not make any strong predictions concerning these. Although intuitively it might seem that decreased precision in beliefs about transitions would lead to an increased rate of alternation (and would if the sensory data really were alternating), it instead leads to faster accumulation of uncertainty about the suppressed stimulus. Differences in the rate of alternation between different sorts of stimuli could be explained through an appeal to deep temporal generative models (Kiebel *et al.* 2009; Friston *et al.* 2017d) that include a hierarchy of temporal scales. As the hierarchy is ascended, time-courses tend to be extended, such that slowly varying representations are housed at the highest levels (Hasson *et al.* 2008; Murray *et al.* 2014). This is important for processes such as working memory (Parr and Friston 2017d), which involve the maintenance of a representation over a time-period that exceeds that of the stimulus presentation (Funahashi *et al.* 1989). In the present context, this also means that the level at which covert attention is deployed in a generative model will influence the frequency with which the stimulus appears to alternate. For natural visual images used in rivalry paradigms, there will be several hierarchical levels in play (Yuille and Kersten 2006). Given that conditions such as autism are associated with an altered balance between prior and likelihood precision, the influence of each hierarchical level on its adjacent levels is altered. Excessive precision ascribed to lower (faster) levels of the hierarchy might then mean a greater rate of perceptual alteration than when this precision is greater at higher (slower) levels. This predicts that functional imaging of rivalry would reveal greater involvement of lower-level sensory cortical regions in individuals with a faster rate of perceptual alternation, and of higher-order sensory or association cortices in those exhibiting slower rates of alternation. This must be nuanced a little by considering the *relative* precisions between levels, as a low precision linking a higher to a lower level manifests as a weak (empirical) prior for the lower level, and a *relatively* high precision ascribed to ascending input to the lower level. In this scenario, there is a form of functional disconnection between the two levels that could lead to increased activity in the lower region without driving changes in the percept at higher levels. This predicts the opposite of the above. These opposing predictions might provide the means to disambiguate between excessive precision ascribed to a likelihood distribution and insufficient precision associated with a prior distribution.

The model used to generate these simulations is formulated in discrete time, so it is difficult to use this to make any definitive statements about the sorts of distributions we might expect for the associated (continuous) dominance times. An interesting next step in accommodating these sorts of data would be to specify a generative model that generates these data from our simulation results. This could be done relatively simply by starting from an assumed duration for a discrete time-step, and adding noise to this. Naively, we might consider treating our assumed duration as the mode of a Gaussian distribution and adding normally distributed noise. However, the problem with this is that it implies a finite probability for a negative dominance time. A simple solution to this is to assume that the distribution of the logarithm of dominance times is normally distributed. When exponentiated, the resulting distribution will preclude negative dominance times and would have a leptokurtic (heavy-tailed) form consistent with empirical characterizations of these distributions (Levelt 1966; Levelt 1967).

## Discussion

In the above, we simulated two paradigms that pose difficulties for passive Bayesian accounts of brain function. Through simulations of Troxler fading and binocular rivalry, we demonstrated how appealing to (covert and overt) active inferential processes renders these counterintuitive perceptual phenomena emergent properties of variational inference. To explain these processes, we appealed to the idea of a generative model that makes predictions about sensory data, and that can be used to explain incoming data in terms of their causes. The generative models used above combine two key features. The first is that action (whether skeletomotor or mental) may be used to forage for the data that best resolves uncertainty about a scene. The second feature is a prior belief that the world is not deterministic, but changes over time with a certain probability. Together, these ensure the accumulation of uncertainty about unattended stimuli that are balanced by sequential sampling to resolve this uncertainty. By using the same features to account for multiple perceptual phenomena, we not only comply with Occam’s razor but advance empirically testable hypotheses about the relationship between these paradigms. In brief, those individuals who report a longer time until the stimuli have faded in a Troxler paradigm should exhibit less belief updating (and its associated electrophysiological manifestations) at perceptual transitions in a binocular rivalry paradigm.

A possible objection to these ideas comes from experiments based upon retinal image stabilization. These attempt to maintain an image in exactly the same place on the retina, ensuring the image is always visible in the same retinotopic location and is invariant to eye movements. Although always within the field of view, these images appear to fade over time (Yarbus 1960). The idea that repeated fixations are required to maintain a percept of a (potentially) volatile stimulus appears to contrast with this finding. The apparent contradiction here may be resolved by appealing back to the idea of perceptions as hypotheses (Gregory 1980). This perspective says that we do not perceive the visual data our retina receives, but the inferences drawn from these data. If something is stabilized relative to the retina, then the sensory data it causes are invariant to hypotheses about the external causes of visual data, and the (saccadic) experiments used to resolve uncertainty about these hypotheses (Rozhkova and Nikolaev 2015). As such they cannot be used to disambiguate between perceptual hypotheses. A related interpretation of this phenomenon appeals to the idea that it is itself an example of binocular rivalry. Under this view, there is always a greater potential information gain by attending away from the fixed image, and to the stimuli visible using the other eye instead. This would reproduce the pattern of Fig. 7B, where the uninformative stimulus rapidly fades. This is highly consistent with the failure to elicit fading when the same image is stabilized for both eyes (Rozhkova *et al.* 1982).

Another possible objection to the model used for the Troxler fading example is that an eye movement, no matter where it is to, might yield information about all of the stimuli. This could be motivated in terms of the magnocellular signal arising from the periphery of the retina (Livingstone and Hubel 1988; Zeki and Shipp 1988; Zeki and Shipp 1989; Nealey and Maunsell 1994). Cells contributing to these pathways respond to high-frequency temporal information, so it could be argued that any action will induce high-frequency temporal changes in the retinal periphery that would provide information about all of the stimuli. If this is true, this does not detract from the notion that performing eye movements provides uncertainty resolving information. It simply suggests that there is less specificity in which eye movement is performed. However, there are good reasons to think that the specific eye movements are important. First, the peripheral retina and magnocellular system have very low colour sensitivity. As long as the luminance of the stimuli is matched to the background, the parvocellular pathways originating from the fovea should be much more important (Bachy and Zaidi 2014). Second, saccadic suppression (Bridgeman *et al.* 1975) limits the communication of visual data through the visual system during self-generated eye movements, suggesting that it is the data garnered during fixations that are used to constrain perceptual inference. Third, the importance of foveation is illustrated in psychophysical data investigating the relationship between stimulus eccentricity and fading (Lou 1999; Bachy and Zaidi 2014). Faster fading of more eccentric stimuli implies that the choice of fixation location is important and is consistent with the reduced precision associated with data from the retinal periphery relative to the fovea.

We have assumed in the above that estimates of the transition precision remain constant throughout. However, it is highly probable that this is something updated over time, inferred from sensory data. For example simulations of how this sort of updating may proceed using this sort of model, please see Parr *et al.* (2018). This implies fluctuations in these precisions which, if encoded by chemicals such as noradrenaline, could manifest through changes in pupillary diameter over time. The time-course of this updating may be important when perceptual alternation occurs, and this may be an important direction for future work (Einhäuser *et al.* 2008, 2010; Naber *et al.* 2011). It additionally offers another avenue by which the relationship between rivalry and perceptual fading may relate to each other that depends upon objective physiological measurements. This could complement the psychophysical and electrophysiological approaches suggested above.

Our account of attention as (covert) action resonates with previous research on the role of attention in binocular rivalry. Although binocular rivalry is highly resistant to volitional control, percept dominance and alternation rate are both sensitive to endogenous attentional allocation (Lack 1974; Ooi and He 1999; Meng and Tong 2004; Chong *et al.* 2005; van Ee *et al.* 2005; Paffen *et al.* 2006; Alais *et al.* 2010). Such findings are accommodated by the active inference framework, where selective attention is understood as gain control (i.e. descending predictions about the precision of a stimulus). The simulations presented above speak to a rather more subtle, involuntary attentional mechanism; one which *drives* (rather than modulates) binocular rivalry. Such nonconscious inferential processes could also underwrite various (e.g. affective) factors known to bias rivalrous perceptual experience towards ‘salient’ stimuli (Alpes and Gerdes 2007; Bannerman *et al.* 2008; Marx and Einhäuser 2015). Salient stimuli afford greater opportunities for the resolution of uncertainty and are thus preferentially sampled (this is equivalent to favouring the more precise stimulus in Fig. 7C). Although gain control and salience ascription rest on neurobiologically distinct mechanisms, they are complementary and deeply interwoven modes of uncertainty reduction under the active inference framework (Parr and Friston 2017d, 2019).

**Figure 8.**
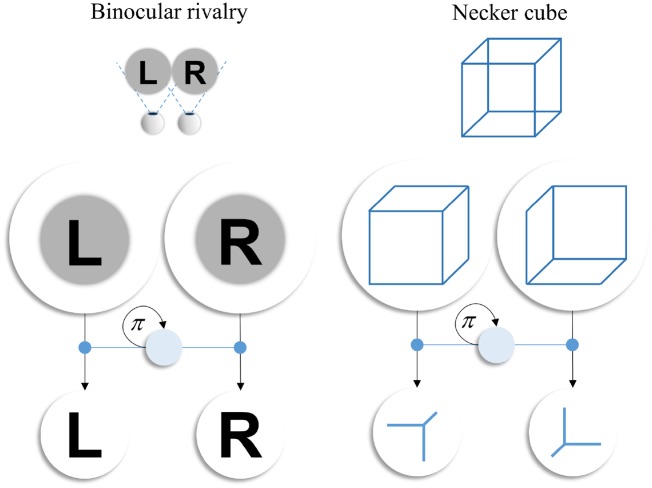
Multi-stable perception. The schematic shown above illustrates how the mechanisms we have employed to simulate binocular rivalry may generalize to other forms of multi-stable perception. This uses the Necker cube as an illustrative example but could be applied to other paradigms. On the left, we show the key features of the generative model used for the rivalry simulation. There are two hidden state factors that represent the presence or absence of ‘L’ and of ‘R’. These generate (black vertical arrows) outcomes that are informative about the presence or absence of each of these visual features. The mapping from the states to their respective outcomes may be very precise or imprecise, depending upon the allocation of attention (a third hidden state factor shown in pale blue), which itself depends upon the choice of policy (*π*). This selects which of the two likelihood mappings is precise. On the right, we illustrate how changing the labels of each of these states and outcomes (without changing the generative model itself) lets us reinterpret the simulation results above in terms of the multi-stable perception associated with a Necker cube. This implies a common architecture for the neuronal message passing, even if implemented in different neuroanatomical structures (Loued-Khenissi *et al.* 2019).

Although we have cast the model here in terms of binocular rivalry, the computational mechanisms generalize to other forms of multi-stable perception. An important example of this is the Necker cube (Necker 1832; Orbach *et al.* 1963), an ambiguous image that depicts the edges of a cube, and is equally consistent with a view of the cube from above or below. As in binocular rivalry, this leads to an alternation in perceiving the cube in each alternative configuration. One way to interpret this is that, when we view a cubic structure, one of the vertices is normally hidden from view by the rest of the cube. This means that one of the vertices that could be attended affords evidence to the hypothesis that the cube is oriented one way, whereas another affords evidence to an alternative hypothesis. Based on this, we could use the same generative model as used to simulate binocular rivalry to simulate the experience of multi-stable perception induced by a Necker cube [which could also include overt eye movements (Einhäuser *et al.* 2004)]. As illustrated in Fig. 8, this would mean replacing the hidden states representing the presence or absence of ‘R’ and ‘L’ with analogous states for presence or absence of a cube viewed from above and below. The outcomes are the associated vertex of each of the two cubes. The attentional state is the same, allowing the selection of a precise likelihood mapping between each cube and the presence or absence of its associated vertex. The above makes the point that the interpretation of the simulations presented above in terms of binocular rivalry rest upon the labels we have assigned to the states and outcomes. Simply changing these generalizes the computational mechanism to other forms of multi-stable perception.

Interestingly, the frequency of perceptual transitions for the Necker cube may be manipulated by physically alternating presentation of the cube and a blank stimulus at different frequencies. Perceptual transitions occur with maximal frequency when the stimulus is presented at a rate of about 2 Hz (Orbach *et al.* 1966). Given the blank periods in between, this means visual input undergoes a transition at about 4 Hz. This coincides with the theta frequency range associated with saccadic sampling and perceptual sequences. In other words, at this rate, every discrete time-step is aligned with a transition in the real stimulus, implying a generative model where things change at every step. As we move away from this frequency of stimulus presentation (either increased or decreased), this is no longer the case, and we would expect a reduced frequency of perceptual transitions, consistent with empirical observations (Orbach *et al.* 1963).

Although we have focused upon the computational basis for a certain kind of perceptual phenomenology, this also offers a set of constraints upon the neurobiology for these forms of perceptual awareness. Specifically, it suggests that those pathways engaged in communication between sensory and motor regions are crucial in ensuring active solicitation of informative sensations. Anatomically, there are several prominent white matter tracts connecting the motor and sensory regions (Catani *et al.* 2002; Thiebaut de Schotten *et al.* 2005). These structural data are complemented by the engagement of the regions connected by these tracts during functional imaging studies (Büchel *et al.* 1998; Corbetta *et al.* 1998; Ptak and Schnider 2010; Corbetta and Shulman 2011; Fiebelkorn *et al.* 2018). Notably, the same set of frontal and posterior cortical regions seem to be activated by overt (oculomotor) and covert attentional processing. These are typically divided into a symmetrical dorsal frontoparietal network, and a right-lateralized ventral frontoparietal network. It is the latter that has been implicated in imaging studies of rivalry (Lumer *et al.* 1998; Brascamp *et al.* 2018), and that is often implicated in disorders of awareness such as visual neglect.

The generative models appealed to here may be interpreted in terms of this anatomy (Parr and Friston 2018), with frontal regions computing either the fixation location or the best attentional target. These presumably exert a modulatory influence, via the right ventral temporoparietal cortex (Sheinberg and Logothetis 1997), over connections from early visual areas to the ventral visual stream in the temporal cortex, and reciprocal projections from temporal to occipital areas. Based on this hypothetical association between the computational architecture associated with active inference and the functional anatomy of rivalry, it should be possible to evaluate the processes we have simulated in relation to neuroimaging data using dynamic causal modelling (Friston *et al.* 2003). We hypothesize that the effective connectivity between occipital cortices and ventral visual regions representing alternative stimuli (e.g. faces and houses) during rivalry should be modulated whenever a perceptual switch occurs. This modulation should be driven by changes in ventral frontal cortical regions (Weilnhammer *et al.* 2013, 2017). It is worth pointing out that changes in early visual areas, including the lateral geniculate nucleus (Haynes *et al.* 2005; Wunderlich *et al.* 2005) and primary visual cortex (Polonsky *et al.* 2000), have also been demonstrated in the rivalry. These findings endorse the notion of reciprocal message passing, as they suggest that changes in higher cortical regions are propagated to lower cortical and subcortical regions as descending predictions. In the future, the models outlined here should be extended to hierarchical models (Friston *et al.* 2017c) that try to predict not just the abstract features associated with a stimulus, but also the lower-level visual features that might be represented in earlier parts of the visual pathway. Associating these models with the anatomical structures that implement them offers the opportunity to interpret findings from neuroimaging of perceptual awareness (Rees 2001, 2007; Brascamp *et al.* 2018) in terms of the computational processes that underwrite them.

## Conclusion

Visual illusions have often been used in the study of perceptual awareness, as they reveal the importance of internally generated perceptual content when presented with ambiguous stimuli. Typically, models of these processes have implicitly assumed that the brain is a passive recipient of data that engages in inferential processes to try to explain these data. Here, we have focused upon two perceptual phenomena that largely elude this approach. By framing these in terms of the active processes that underwrite engagement with a (potentially) dynamic environment, we find that Troxler fading and perceptual alternation – in the presence of rivalrous stimuli – emerge from the accumulation of uncertainty in beliefs about unattended stimuli, and the drive to act to resolve this uncertainty. Equating elements of the computational mechanisms that underwrite these processes suggests several directions for further empirical investigation, both in terms of the association between an individual’s experience of each of these paradigms, and the functional anatomy that supports these. Ultimately, we hope that this will afford an opportunity to better understand the pathological computations that give rise to abnormalities of perceptual awareness, such as autism, psychosis and visual neglect.

## Data Availability Statement

The simulations presented in this article were performed using standard software routines (here **spm_MDP_VB_X.m**) with the generative models specified as described in the text and figures. These routines are available as Matlab code in the SPM academic software: http://www.fil.ion.ucl.ac.uk/spm/. Simulations of the sort reported above can be reproduced (and customized) via a graphical user interface by typing in ≫**DEM** and selecting the ‘visual foraging’ demo.
